# Supplementary material for: Development and Validation of Indirect Enzyme-Linked Immunosorbent Assays for Detecting Antibodies to SARS-CoV-2 in Cattle, Swine, and Chicken
Source: Viruses. 2022 Jun 22;14(7):1358. doi: 10.3390/v14071358 (PMC9317974; doi:10.3390/v14071358)
Supplement: Supplementary file 1 [file viruses-14-01358-s001.zip › viruses-1716863-supplementary.pdf]

viruses MDPI

Article

Development and Validation of Indirect Enzyme-Linked Immunosorbent Assays for Detecting Antibodies to SARS-CoV-2 in Cattle, Swine, and Chicken

Abhinay Gontu [1,2], Erika A. Marlin [1,3], Santhamani Ramasamy [1], Sabarinath Neerukonda [4], Gayatri Anil [1], Jasmine Morgan [1], Meysoon Quraishi [1], Chen Chen [5], Veda Sheersh Boorla [5], Ruth H. Nissly [2], Padmaja Jakka [1,2], Shubhada K. Chothe [1], Abirami Ravichandran [6], Nishitha Kodali [1,7,] Saranya Amirthalingam [1,7], Lindsey LaBella [1], Kathleen Kelly [2], Pazhanivel Natesan [8], Allen M. Minns [7,9], Randall M. Rossi [7], Jacob R. Werner [10], Ernest Hovingh [1], Scott E. Lindner [7,9], Deepanker Tewari [11], Vivek Kapur [7,10,12], Kurt J. Vandegrift [7,12,13], Costas D. Maranas [5], Meera Surendran Nair [1,2,*] and Suresh V. Kuchipudi [1,2,7,12,*]

1 Department of Veterinary and Biomedical Sciences, The Pennsylvania State University, University Park, PA 16802, USA; abhinay@psu.edu (A.G.); erika.marlin@pfizer.com (E.A.M.); sqr5895@psu.edu (S.R.); gxa5148@psu.edu (G.A.); jkm5870@psu.edu (J.M.); mjq5073@psu.edu (M.Q.); padmaja@psu.edu (P.J.); skc172@psu.edu (S.K.C.); nkk5370@psu.edu (N.K.); ska5899@psu.edu (S.A.); lcl122@psu.edu (L.L.); eph1@psu.edu (E.H.)
2 Animal Diagnostic Laboratory, Department of Veterinary and Biomedical Sciences, The Pennsylvania State University, University Park, PA 16802, USA; rah38@psu.edu (R.H.N.); kmk6898@psu.edu (K.K.)
3 Clinical & Diagnostic Assay Development Group, Pfizer, Pearl River, NY 10965, USA
4 U.S. Department of Health and Human Services, Silver Spring, MD 20993, USA; nnvsnath@gmail.com
5 Department of Chemical Engineering, The Pennsylvania State University, University Park, PA 16802, USA; czc325@psu.edu (C.C.); vqb5186@psu.edu (V.S.B.); cdm8@psu.edu (C.D.M.)
6 Department of Integrative and Biomedical Physiology, The Pennsylvania State University, University Park, PA 16802, USA; aur1121@psu.edu
7 Huck Institute of Life Sciences, The Pennsylvania State University, University Park, PA 16802, USA; amm504@psu.edu (A.M.M.); rmr29@psu.edu (R.M.R.); sel27@psu.edu (S.E.L.); vxk1@psu.edu (V.K.); kjv1@psu.edu (K.J.V.)
8 Madras Veterinary College, Tamil Nadu Veterinary and Animal Sciences University, Chennai 600007, India; drnpvel@gmail.com
9 Department of Biochemistry and Molecular Biology, The Pennsylvania State University, University Park, PA 16802, USA
10 Department of Animal Science, The Pennsylvania State University, University Park, PA 16802, USA; jrw140@psu.edu
11 Pennsylvania Department of Agriculture, Pennsylvania Veterinary Laboratory, Harrisburg, PA 17110, USA; dtewari@pa.gov
12 Center for Infectious Disease Dynamics, The Pennsylvania State University, University Park, PA 16802, USA
13 Department of Biology, The Pennsylvania State University, University Park, PA 16802, USA
* Correspondence: mms7306@psu.edu (M.S.N.); skuchipudi@psu.edu (S.V.K.)

SARS-CoV-2 Spike Pseudovirus Production and Neutralization Assay

SARS-CoV-2 pseudo viruses were produced as described previously by Crawford et al [55,56]. Plasmids encoding spike proteins of SARS-CoV-2 Wuhan strain and Alpha, Beta, Gamma, Delta, and Omicron VOCs were co-transfected with lentiviral backbone plasmid expressing firefly luciferase and lentiviral helper plasmids expressing HIV Gag-pol, Tat and Rev. 293T cells were transfected with the plasmids using jetPRIME® transfection reagent (Polyplus®). Supernatants containing pseudo-typed particles were collected at 48 hr post-transfection and filtered with a 0.45 µM filter. For neutralization assays, HIS produced against RBD in cattle, swine and chicken was 3-fold serially diluted prior to incubation with pseudo viruses for one hr at 37°C. Pseudovirus/HIS mixtures were then added to 96-wells pre-seeded with 293T-hACE2/TMPRSS2 cells (NR-55293, BEI resources). Luciferase activity was measured at 48hr post infection to quantify the neutralization titer of tested sera.

Table S1. Characteristics of species-specific iELISAs developed for detecting antibodies against SARS-CoV-2.

Characteristic	Value (95% Confidence Interval)
**Cattle**	
**Sensitivity**	100.00% (75.29% to 100.00%)
**Specificity**	100.00% (97.57% to 100.00%)
**Accuracy**	100.00% (97.76% to 100.00%)
**Swine**	
**Sensitivity**	100.00% (54.07% to 100.00%)
**Specificity**	100.00% (97.57% to 100.00%)
**Accuracy**	100.00% (97.66% to 100.00%)
**Chicken**	
**Sensitivity**	100.00% (85.75% to 100.00%)
**Specificity**	100.00% (97.57% to 100.00%)
**Accuracy**	100.00% (97.90% to 100.00%)

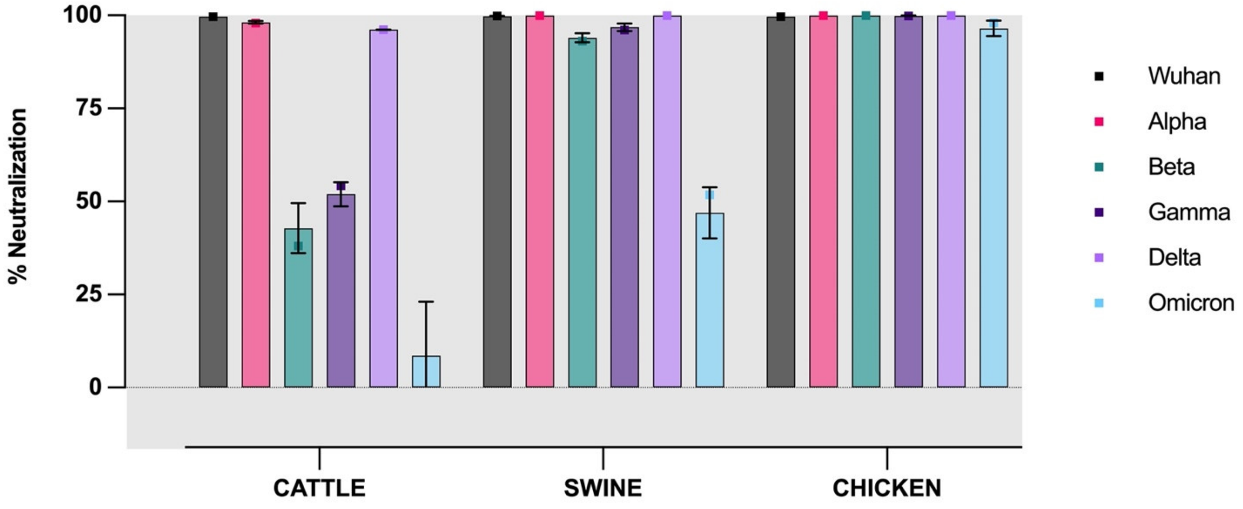

Figure S1. Hyperimmune serum raised with Wuhan RBD cross reacts with SARS-CoV-2 VOCs. Cattle, swine, and chicken hyperimmune sera raised against SARS-CoV-2 RBD were tested for the ability to neutralize SARS-CoV-2 pseudoviruses expressing spike protein of Wuhan and Alpha, Beta, Gamma, Delta and Omicron VOCs. The hyperimmune serum from all three species broadly cross-neutralized all the tested SARS-CoV-2 VOCs. Height of bar represents the percent (%) neutralization. Error bars represent the standard deviation.
